# Literature Review of the Implications of Exercise Rehabilitation Strategies for SARS Patients on the Recovery of COVID-19 Patients

**DOI:** 10.3390/healthcare9050590

**Published:** 2021-05-18

**Authors:** Wei Cui, Ting Ouyang, Ye Qiu, Di Cui

**Affiliations:** 1 Department of Physical Education, Hunan University, Changsha 410000, China; cuiwei@hnu.edu.cn (W.C.); ting_ouyang@163.com (T.O.); 2 College of Biology, Hunan University, Changsha 410000, China; qiuye@hnu.edu.cn

**Keywords:** SARS-CoV-2, COVID-19, convalescent patient, clinical symptoms, exercise rehabilitation, integration of sports and medicine

## Abstract

As a global pandemic, COVID-19 shows no sign of letting up. With the control of the epidemic in China, the proportion of patients with severe and critical diseases being cured and discharged from hospital has increased, and the recovery of COVID-19 patients has become an important issue that urgently needs attention and solutions. By summarizing the exercise rehabilitation strategies and progress of SARS in 2003, this paper analyzed the differences in clinical indicators and recovery characteristics of severe pneumonia caused by the two viruses, and provided comprehensive exercise guidance and intervention strategies for COVID-19 patients for rehabilitation and nursing by referring to the problems and treatment strategies in the rehabilitation and nursing work of SARS. In the post-epidemic period, China will build a multi-dimensional epidemic prevention system by improving the effectiveness of mass training and strengthening local risk prevention and control. This paper discusses the exercise rehabilitation strategy of SARS patients after recovery, which has guiding significance for exercise intervention and scientific fitness of COVID-19 patients after recovery during epidemic prevention period.

## 1. Introduction

COVID-19 has become a serious global epidemic, with “second wave” outbreaks occurring in some countries that have not been properly controlled. In January 2020, the World Health Organization (WHO) also declared that new outbreaks are expected to last longer and require longer preparation, calling the COVID-19 outbreak a “public health emergency of international concern.” [1]. China has set a successful example for the rest of the world in the prevention and control of COVID-19. Since the emergence of COVID-19 in Wuhan at the end of 2019, the epidemic has rapidly spread to all provinces across the country, attracting great attention from local governments and health departments at all levels. COVID-19 has caused a huge burden on the medical system and caused a huge impact on the normal life order of the public [2,3,4]. At present, the prevention and control of the epidemic in China has entered the later stage. With the increasing proportion of patients cured and discharged from hospital, although clinical treatment has ended, their organ functions have not yet returned to normal. Therefore, continuous attention should be paid to the recovery of patients after recovery and regular rehabilitation training should be accepted to assist treatment. At present, there are still many questions and uncertainties about the transmission, infection and treatment of COVID-19, and the best way to prevent and control COVID-19 is to prevent infection. Studies have confirmed [5] that physical exercise can enhance the body’s immune capacity, reduce the risk of viral infection, and have a positive effect on the prevention of COVID-19 infection. By comparing the etiology, pathogenesis, clinical characteristics, prevention and treatment of SARS and COVID-19 pneumonia, it is confirmed that the disease and secondary systemic inflammation caused by the two kinds of coronaviruses are similar [6,7,8]. According to the situation of acute respiratory syndrome caused by coronavirus around 17 years, the problems and countermeasures in the rehabilitation and nursing work of SARS were summarized, so as to provide reference for the exercise intervention of COVID-19 patients after recovery during the epidemic prevention and control period, and provide valuable ideas for promoting scientific exercise and sports epidemic prevention for the whole people.

## 2. Basic Overview of COVID-19

**Figure 1:**
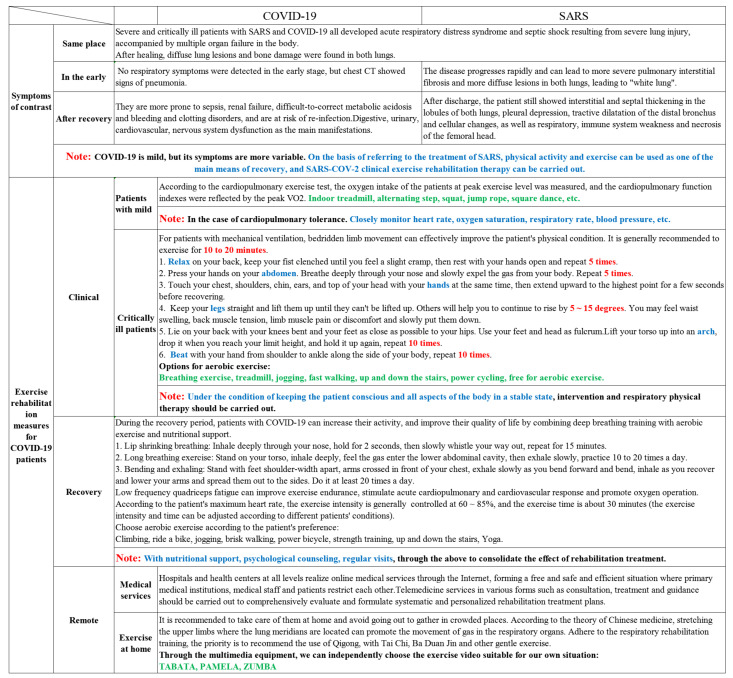
The comparison of symptoms caused by COVID-19 and SARS and implication on rehabilitation strategies of COVID-19 in China.

On 12 January 2020, the World Health Organization (WHO) initially named the 2019 novel Coronavirus (2019-nCoV) which erupted in Wuhan, and then the International Committee for Classification of Viruses classified it as Severe Acute Respiratory Syndrome Coronavirus 2 (SARS-CoV-2) and the WHO officially named the disease caused by SARS-CoV-2 as COVID-19 [9,10]. The novel β-coronavirus SARS-CoV-2 has a high homology with SARS-CoV, Both of them use angiotensin-converting enzyme 2 (ACE2) as host receptor, and invading human cells and causing the same damage means that they may cause similar symptoms in humans [6,11]. Fever, dry cough and fatigue were the main clinical manifestations in all patients. Acute respiratory distress syndrome and septic shock occurred in severe patients, and organ failure occurred in all patients. After recovery, lung diffuse lesions and bone damage occurred in all patients [12,13,14,15,16,17]. Figure 1 summarizes the clinical characteristics and prognosis of patients with COVID-19 and SARS. In summary, the COVID-19 illness condition was milder while complications were more variable. These results were consistent with the clinical characteristics analysis results of 1099 COVID-19 patients from 552 hospitals in more than 30 provinces in China extracted by Guan et al. [18] from 11 December 2019 solstice to 29 January 2020, Huang et al. [19] of 41 confirmed COVID-19 cases and Wang et al. [20] of 138 confirmed COVID-19 cases. The clinical data showed that the elderly patients with hypertension, chronic obstructive pulmonary disease, diabetes and other basic diseases had a higher risk of SARS-CoV-2 infection, which was easy to deteriorate after infection, and even result in death [20].

### 2.1. Mode of Transmission

The main mode of transmission of SARS-CoV is close contact with droplets or with respiratory secretions of patients [21]. SARS-CoV-2 virus is transmitted mainly by contact, droplets and aerosols, while asymptomatic infected persons cannot be ignored in the transmission process [22,23]. Researchers also detected the virus in the feces of confirmed patients, suggesting that SARS-CoV-2 can replicate and survive in the digestive tract and suggesting the possibility of fecal-oral transmission [24]. It has been reported that the nucleic acid test in the throat swab of the newborn 30 hours after the mother’s diagnosis was positive, suggesting the possibility of mother-to-child transmission [25].

### 2.2. Treatment Countermeasures

On 19 August 2020, China’s National Health Commission officially released the COVID-19 Treatment protocol (trial eighth edition) [26] and on 14 January 2021, the US National Institutes of Health (NIH) issued the COVID-19 Treatment Guidelines [27]. The overall treatment principle of the two is the same, with effective antibody-virus, immunosuppression, hormone therapy, as well as the corresponding organ support therapy to improve the patient’s symptoms. 

At present, the clinical treatment strategy for COVID-19 is relatively conservative, and there is no highly effective treatment. 90% of the cured patients are mild patients, which mainly rely on the patients’ own immunity [28].

Studies have shown that plasma therapy is well tolerated in patients with severe COVID-19 during convalescence and can neutralize viremia in patients with severe COVID-19, with no serious adverse reactions observed [29]. Prophylaxis of anticoagulants is recommended for patients at risk of thromboembolisis [30]. In conclusion, treating basic diseases, actively preventing and treating complications, and preventing secondary infections after treatment are the key points for COVID-19 treatment [31,32].

Based on the experience of the treatment of SARS, physical activity and exercise treatment could be applied as main methods of rehabilitation to carry out research on clinical sports rehabilitation of SARS-CoV-2 epidemic to provide comprehensive rehabilitation exercise guidance and ideal therapy for COVID-19 patients. The epidemic situation of SARS is different from the outbreak of COVID-19, and various places have achieved certain results in the prevention and control of health work. Measures have been taken to control social distance, close entertainment places, prohibit gathering activities, study and work at home, avoid close contact, and choose home isolation. This situation forces people to change the way they exercise. At the same time, the lack of home rehabilitation equipment for people with diseases leads to a significant decline in physical activity for people of all ages due to limited activity space and unexpected long duration inactivity. The unscientific exercise behavior of COVID-19 patients also increases the risk of heart damage and cardiogenic death during exercise. Therefore, we urgently need to summarize and reflect from the rehabilitation and nursing work of SARS, and put forward solutions to improve our response to the impact of public health emergencies on people’s work, study and life [33,34].

### 2.3. COVID-19 Vaccine

For the prevention and control of this kind of disease, on 24 January 2020, China’s Center for Disease Control and Prevention successfully isolated the first novel coronavirus virus strain in China. On 19 June the same year, China’s first new coronavirus mRNA vaccine was approved to start the clinical trial. By 25 February 2021, the number of new coronavirus vaccines eligible for the market in China had reached 4, including 3 inactivated vaccines and 1 adenovirus vector vaccine. As of 24:00 on 20 March 2021, more than 70,000 doses have been vaccinated nationwide, and no serious adverse reactions have been reported [35].

## 3. The Research Methods

On the basis of literature survey, the current research status of COVID-19 at home and abroad was summarized. To reveal the main achievements and development trends related to COVID-19 rehabilitation nursing at the present stage. In this paper, the author takes into account the requirement of comprehensiveness and high quality of literature materials, and selects SCI, SSCI, EI from the core collection of Web of Science and Core, CSSCI, and CSCD academic journals from the advanced retrieval of CNKI. A total of 2517 literatures were retrieved and deleted, among which 132 literatures were compared with specific national conditions of China for reference.

### 3.1. The Data Source

This paper retrieved Chinese and English periodical materials from two databases for visual analysis. Respectively these are: (1) In the advanced search mode of China National Knowledge Network (CNKI), Chinese journal libraries of SCI sources, EI sources, core journals, CSSCI and CSCD were selected, and a total of 164 journal articles from 2003 to 2021 were retrieved with “SARS” and “rehabilitation” as the topic of fuzzy search, and the “co-contained” relationship was used to link fuzzy matching. A total of 89 results with the topic or title of “SARS rehabilitation” were selected, and the published years were mainly from 2003 to 2006. After manual review, the remaining 61 journal articles were selected as the final analysis objects after eliminating the irrelevant sample literatures. (2). In the basic retrieval mode of Web of Science, a total of 55 journal articles from 2002 to 2021 were retrieved with the theme of “SARS Rehabilitation” as the core collection of MEDLINE and Web of Science. The years of publication were mainly from 2003 to 2005, and the remaining 43 English journal articles were selected as the final analysis objects after eliminating the relevant literatures through manual browsing and screening review.

### 3.2. Research Topics

The study focused on rehabilitation care and exercise prescription related to COVID-19. The content covers the rehabilitation and exercise of SARS patients, the rehabilitation of respiratory muscle, the improvement of athletic ability, mental health and medical public health. Keywords can reflect the research object of the article, research methods and so on, the keywords of the literature of sample extraction, which can effectively discover the change characteristic of the field and development path, on the theme of SARS rehabilitation retrieval process, according to the different expression of synonyms, extension, in both English and Chinese abbreviations, and so on and so forth, statistical data of keywords. Top 10 keywords: “recovered patients”, “lung function damage”, “magnetic resonance imaging”, “osteonecrosis”, “pulmonary fibrosis”, “lung diffusion function”, “osteoporosis”, “forced vital capacity”, “mental function”. Through keyword retrieval and multivariate data integration, it is helpful to realize the management of literature materials, and more accurate to review and discuss the data that have been mastered.

### 3.3. Document Management

Quantitative method is used to analyze the data. Web of Science searches topics including rehabilitation, exercise, physical activity, movement, walking, gymnastics, aerobics, resistance, games, stretching, yoga, tai chi, tai chi, qigong and a variety of ball games. The CNKI retrieval method is as follows: the subject word is “COVID-19” and includes “Recovery”, and the retrieval period is from 2019 to 2021, until 25 April 2021.A total of 2704 literatures were retrieved, 84 of which were compared with China’s specific conditions for reference. Big data analysis method was used to compare the COVID-19 COVID-19 situation, patients’ condition, recovery degree, follow-up treatment, home isolation and other data from the perspectives of total volume, time and space. From the perspective of macroeconomic benefits, this paper evaluates China’s medical and health industry in the post-epidemic era and puts forward a situation that suits the national conditions and is easy to manage.

## 4. Patients with COVID-19 Were Treated with SARS Rehabilitation Protocol

### 4.1. Respiratory Function

In respiratory rehabilitation training, posture management, good training, chest relaxation training, activity and exercise training play an important role in improving respiratory function of SARS patients, key techniques such as preventing the harm of drugs and other complications caused by long-term bed rest, and avoiding virus infection. Huang’s team [36,37] investigated the convalescence of 21 workers who had been infected with SARS, and found that the convalescence symptoms of SARS patients included mild cough and sputum, suffocation, fatigue and other symptoms, short breath, quiet heart rate, and abnormal lung diffusion function and arterial blood oxygen partial pressure. In the course of 75 days, SARS patients were given rehabilitation therapy for 3 weeks, with deep breathing exercise as the main method, combined with body flexibility exercise, lasting 8 to 10 min, doing 2 to 4 times a day, which can effectively improve the abnormal lung diffusion function, increase lung compliance, improve lung air capacity, prolong deep breathing time, and ensure adequate air exchange [38,39]. After 3 weeks of rehabilitation treatment, the abnormal lung diffusion function of SARS patients in the respiratory department of Peking University Hospital was 100% significantly improved, and the adverse symptoms of respiratory function during the recovery period were significantly improved compared with the patients without rehabilitation treatment during the same period. Nan et al. [40] showed that ultrasound is usually used in SARS patients to change the pathology of lung function injury after clinical rehabilitation. Through direct current iodide ion back and chest physical factor treatment, once a day, 15~20 minutes each time. It can improve breathing difficulties, reduce pulmonary interstitial disease clinical manifestations, and maximum limit promote the recovery of lung function is perfect [41]. Rehabilitation of physical factors is the necessary measures to improve the quality of life.

For patients with COVID-19, ventilator exercise can be used to improve lung function and dyspnea symptoms such as CO_2_ hyperventilation, diaphragm pacing, breathing training equipment, etc. The simplest is blowing balloons or candles, that is, after deep inhalation, blowing balloons and candles, until the feeling stops. The number and time of exercise are determined by the patient’s own state.

### 4.2. Sports Ability

During the SARS epidemic, the death rate of elderly patients over 60 is more than 50% [6,7,8]. Novel coronavirus is widely susceptible to infection, with patients ranging in age from 25 to 89 years, most concentrated in 35 to 55 years, and fewer cases of confirmed infection in children and infants.

In order to control the spread of SARS-CoV and the development of the epidemic, glucocorticoids were widely used in the emergency treatment of SARS, so many patients developed symptoms of femoral head necrosis, as well as pulmonary dysfunction and other side effects of drugs. As one of the main means of pulmonary rehabilitation training, sports rehabilitation was of great significance for the improvement and recovery of patients with acute or chronic lung injury. Pulmonary function was one of the most important detection methods to evaluate the degree of disease, clinical symptoms, prescription efficacy, prognosis recovery and psychological status. In the clinical work of rehabilitation, a large number of patients who are bedridden or receive mechanical respiration should receive rehabilitation treatment based on safety assessment. Lau‘s team [42] recruited 133 patients recovering from SARS that only received physical therapy to observe the cardiopulmonary and skeletal muscle performance of the patients. Physical improvement was evaluated through walking, strength training, tummy and push-up tests over a six-week period, and the results showed that the exercise training program was effective in improving the cardiorespiratory and skeletal muscle health of SARS patients. In the protection of life under the condition of steady state stable, to increase the chance of sit down and rise up, the bed movement and activities were widely applied in clinical, letting patients facilitate from passive to accept early motion to independently complete the movement; the rehabilitation exercise positively improved the disease condition in patients with early, middle, critical period, and disease after preoperative, postoperative and the outcome of the entire process. SARS patients generally had mechanical bed rest treatment for about one month, and the main symptoms in the convalescent period were fatigue, easy fatigue, fast resting heart rate, and obvious shortness of breath after light exercise, which were closely related to the damage of bed rest and the disease itself to the body, especially the lung function damage. In the recovery period of most SARS patients, systemic joint pain symptoms appeared, and bone ischemia and necrosis was relatively common, and its prevalence was greatly related to the excessive use of hormones during treatment [43,44]. Case observation of nine medical workers infected with SARS in Dongzhimen Hospital of Beijing University of Chinese Medicine, showed that hormone-induced femoral head necrosis usually manifested as kidney deficiency. According to Wang [45], poor renal function could lead to a poor blood flow and lead to ischemic necrosis of the femoral head. Oral Chinese medicine intake, external Chinese medicine foot soaking, physiotherapy and hyperbaric oxygen therapy could relieve blood vessel pressure, improve microcirculation, accelerate tissue repair, and increase blood oxygen tension. During rehabilitation period, knee extension/flexor muscle strength exercise and light aerobic exercise had a good effect on patients’ recovery. Through peripheral and central effects, physical strength could be enhanced, oxygen utilization efficiency of skeletal muscle also could be improved [46]. For patients with severe lung injury, the peripheral effect should be the main aerobic exercise, supplemented by the central effect. Sub-extreme exercise was reported to reduce the symptoms of palpitation and shortness of breath, improve cardiopulmonary dysfunction, and play a key role in improving cardiac output, stroke volume, vital capacity and lung capacity [47,48]. Shen et al. [49] observed 148 cases of SARS of five hospitals in Guangzhou, and showed that 106 cases of upper extremity ischemia necrosis had used hormones in the treatment of SARS, and a few cases closely related to the cumulative dose of hormone and bone avascular necrosis, whereas 42 cases of osteonecrosis patients who did not apply hormone therapy had no bone necrosis. In patients with abnormal chest X-ray or lung CT, some of them have aggravated symptoms such as chest tightness, shortness of breath and dyspnea after activity, so they could not participate in more vigorous physical activities, and all indicators of life should be monitored at all times [50]. Using acupuncture and moxibustion therapy recommended by He, lung inflammation absorption and pulmonary fibrosis was treated; taking advantage of collection of acupuncture moxibustion gained better effects. This method was implemented in the SARS rehabilitation clinic of Beijing Hospital of Traditional Chinese Medicine, and the symptoms of fatigue of the discharged patients were obviously ameliorated, and the curative effect of improving the health quality of life was evaluated objectively [21].

### 4.3. Psychological Situation

To face the “SARS sequelae” left behind due to treatment and prevention, excessive use of hormones leaded to lifelong pain and sufferings, causing great harm to patients’ body and mind, and psychological treatment was essential. In July 2003, an investigation on the patients who had recovered from SARS in Guangdong Hospital of Traditional Chinese Medicine showed that [51] most of the patients had pulmonary lesions and other sequelae, especially the psychological barriers caused by discrimination. Zhong, an academician, said that psychological problems were the biggest sequelae of SARS [52]. Wang et al. [53] investigated the psychological status of 103 cured SARS patients in Guangzhou after recovery, and the results showed that people’s low awareness of SARS led to patients’ fear and rejection psychology, which had a great impact on social relations and interpersonal communication after recovery. Some patients thought that they would be discriminated by the society after the disease, which would lead to a kind of hostility. At the same time, they were afraid that they would spread the disease to their families and friends, which would also lead to depressive behaviors [54].

According to a private survey, Beijing registered a total of 300 patients with “SARS sequelae”, among whom 80% had left their posts due to illness and 60% had family accidents [55]. Depression was almost a common condition among patients recovering from SARS, and they did not receive timely counseling and treatment, which had a very negative impact on their lives. In July 2003, the survey of 69 SARS survivors of Guangdong hospital, showed that 57.97% of the patients with poor mental state, memory drops, 49.3% of the patients reduced work ability and increased scruples in the future work and life, 40.58% of the patients with easy irritable, 20% of the patients fear or feeling alone, but 100% of the patients got family and friends’ concern and support [56]. To fight against diseases, we should maintain a good mental state, pay attention to mental health, nutritional support, and health protection, establish the determination to overcome the disease, and eliminate anxiety, anxiety, depression and other negative emotions. At the same time, it is also very important for the government to improve some institutional guarantees, establish a complete information tracking file for SARS patients, and keep a close eye on the life and health of the “SARS sequelaa”.

## 5. Implications for COVID-19 Exercise Rehabilitation in China

In combination with the pathological research results of COVID-19 in the medical field, relevant studies have elaborated the main principles of strengthening physical exercise to effectively prevent COVID-19 [57]. First of all, moderate physical exercise can promote blood circulation, let the immune cells timely transport and destroy the virus in the body, which is the main basis for physical exercise you effectively resist and contain coronavirus [58].Secondly, increasing the activity of angiotensin converting enzyme (ACE) in the renin-angiotensin system (RAS) during exercise improves the structural adaptability of coronary artery tree, which can effectively resist SARS-CoV-2 and play a positive role in the mechanism of acute myocardial injury caused by COVID-19 [59]. Third, sedentary living at home can lead to muscle fixation, damage and deterioration of mitochondrial stability, and cause organic and systemic inflammation, which is also an important mechanism of COVID-19 pathogenesis [60]. Finally, the study also confirmed that physical exercise effectively counteracts and reduces the negative effects of COVID-19 on people’s physical and mental health [5,61].

Lung function injury greatly reduced patients’ daily activity ability and fatigue degree, pulmonary rehabilitation training was urgently needed to restore the lung function. Liu et al. [62] conducted a 6-week respiratory exercise intervention and rehabilitation treatment on 36 elderly patients with COVID-19 who were over 65 years old without related basic diseases, and the results showed that respiratory exercise could effectively improve the patients’ respiratory function, quality of life and anxiety, providing an important reference for clinical treatment. Investigations showed that more than 75% of COVID-19 hospitalized patients required oxygen therapy, approximately 54% developed respiratory failure, and more than 30% required mechanical ventilation to maintain pulmonary gas exchange. However, prolonged mechanical ventilation can lead to atrophy of the diaphragm and rapid development of systolic dysfunction, leading to the risk of ventilator (diaphragm) weakness and difficulty in getting COVID-19 patients off the ventilator [63]. Participated in related studies have shown that advance moderate endurance exercise, the infection will be after the coronavirus to accept ventilator support, from the endurance exercise induced by the diaphragm preadaptation benefit, endurance exercise can increase the superoxide dismutase (SOD) in the diaphragmatic muscle mitochondrial protein 2 (SOD2) level and the amount of heat shock protein 72 in cytoplasmic protein, promote the diaphragm of biochemical changes, effectively resist COVID-19 induced sepsis complications related to [63,64,65,66]. One thing the needs to be noted is that, in nursing work, a large number of patients who were bedridden and receiving mechanical breathing needed to receive rehabilitation treatment on the basis of safety assessment. Before the formal implementation of sports rehabilitation training, it was necessary to evaluate the symptoms, sports ability, quality of life, mental state and other aspects. Bansal’s team [67] evaluated the performance of 20 adults with disordered breathing on an incremental cardiopulmonary exercise test. It found that VO_2_max was lower than average and that patients more frequently cited dyspnea as the cause of their exercise limitations. However, due to the specific cultural, historical and geographical background in China, the exercise rehabilitation of COVID-19 had its own requirements and characteristics. Similarly, the illness severity pattern was divided into four groups: asymptomatic infected patients, symptomatic patients isolating at home symptomatic patients admitted to hospital, and symptomatic patients requiring ventilator support in critical care. Here, we concluded COVID-19 patients’ exercise rehabilitation strategies in three stages of clinical treatment, restoration, and remote healthcare, and divided clinical patients into two groups of patients with mild disease, critically ill patients, and list specific exercise rehabilitation strategies recommendations accordingly in Figure 1.

### 5.1. Clinical Exercise Rehabilitation Methods for COVID-19 Patients

Mild patients can receive moderate to low intensity aerobic training under the condition of cardio-respiratory tolerance, that is, 40% to 60% of the maximum heart rate (HRmax) and during the training, the heart rate, blood oxygen saturation, and respiratory rate should be monitored. The choice of exercise mode can be indoor treadmill exercise, alternate steps, stepping, squatting, rope skipping and other aerobic exercises. According to relevant reports, the mobile cabin hospitals in Wuhan organized patients with mild COVID-19 to do square dancing, breathing exercises, eight-section brocade Chi Kung, and Tai Chi exercises, which not only improved the cardiopulmonary function and reduced the incidence of complications, but also relaxed the anxiety of patients [13]. The First Affiliated Hospital of Fujian Medical University designed an exercise program named “Eight Segment Pulmonary Rehabilitation Exercise” to improve the condition of COVID-19 patients and promote the recovery of their lung and body functions [68].

For critically ill patients, it is required to strictly monitor the safe implementation of patients’ respiratory rehabilitation on the basis of conventional treatment, and accurately grasp the perfect timing of intervention according to the patient’s condition. The rehabilitation strategy should focus on the patient’s heart rate, blood pressure, respiration, blood oxygen, heart, brain, lung and other organs and the consciousness level of patients. When patients come to a physiologically stable state, intervention and respiratory physiotherapy can be carried out such as posture management (including bedside swinging, lying and sitting, regular turning over and massaging the body and other posture changes) to stimulate the heart, lungs and blood vessels, improve the patient’s blood oxygen transport capacity, and enhance airway status and breathing [69,70,71].

For patients with mechanical ventilation, the bed body movement can effectively improve the physical condition and ameliorate complications of patients with sepsis. The amplitude and scope of activities need to be adjusted according to the disease conditions and individual differences. Generally, it is recommended to be active for 10 to 20 minutes, and patients with hemiplegia or shortage of muscle strength, shall execute the exercise with the help of the guidance of nursing staffs.

The specific actions of horizontal rehabilitation exercise [72] are as follows:

(1) Relaxation training: lie flat on the bed, relax the whole body muscles, clench fists, feel the muscle tension in the fingers, knuckles, and palms, keep the movement until you feel a slight cramp, then open your hands to relax and rest, repeat five times.
(2) Abdominal breathing training: the patient presses his hands on the abdomen, and exercises with the abdomen when breathing, breathes deeply through the nose until it can’t breathe in, and then slowly and rhythmically expel the gas from the body, repeat five times.
(3) Upper limb elevation training: the patient touches the chest, shoulders, chin, ears, and top of the head with the index finger, and then stretches up to the highest point for a few seconds and then recovers, with both hands at the same time.
(4) Elevating lower limbs training: straighten legs and lift them up until fatigue, assisted by others to continue to rise by 5 to 15 degrees, when the patient feels waist soreness, back muscle tension, slight pain or discomfort, switch to the other leg.
(5) Hip bridge training: bend the knees in the supine position, put your feet close to your hips as much as possible, use your feet and head as the fulcrum, add elbows if necessary, lift up to arch, feel the extension of the waist and back, hips and lower limbs When you reach your limit height, put it down and hold it up again, repeat 10 times.
(6) Meridian patting training: the patient pats along the side of the body from shoulder to ankle in turn, alternating up and down, and repeats 10 times.

### 5.2. Exercise Rehabilitation Methods for COVID-19 Patients after Recovery

COVID-19 inpatients are often accompanied by related basic diseases [73], so it is important to remember to blindly carry out all kinds of respiratory supportive therapy. On the basis of clarifying the internal mechanism of respiratory exercise and COVID-19, it is an important field to strengthen the research on the intervention of respiratory rehabilitation exercise for patients with related basic diseases or induced complications, which needs to be deepened and solved urgently. The specific methods are as follows: (1) respiratory breathing: inhale deeply through your nose, hold for 2 seconds, and then slowly exhale through a whistle, repeat practice for 15 minutes; (2) long breathing exercises: stand up torso, relax the muscles, inhale deeply, feel the air enter the lower abdominal cavity, then exhale slowly, the slower the better, fully feel the process of the air leaving the lungs, trachea, and nasal cavity, practice 10~20 times every day; (3) bend and exhale exercise: stand with your feet shoulder-width apart, cross your arms in front of your chest, exhale slowly when you bend your body forward, and inhale when your arms fall and spread out to both sides when you recover, stick to it every day at least 20 times. Besides, abdominal breathing can be found in a variety of physical and mental exercises, which can enhance respiratory function, stimulate the vagus nerve and produce a relaxation response, including yoga, meditation and Qigong [74,75]. The technical operation of abdominal breathing is very simple. The subjects consciously move the stomach when inhaling, tighten the stomach muscles, and let them fall inward when exhaling with focusing on the breath and avoiding to hold your breath [62]. Before training, set the exercise intensity according to the patient’s maximum heart rate, generally controlled at 60–85%, and the exercise time is about 30 minutes (the exercise intensity and time can be adjusted appropriately according to the condition of different patients). During the exercise, the subjects should pay attention to control the heart rate to avoid dizziness, fatigue, shortness of breath, chest tightness, chest pain and other situations. If those situations happen, the exercise program should be stopped immediately. In severe cases, nitroglycerin and other drugs need to be applied.

By cardiopulmonary exercise test (CPET), in the case of load increment, by breathing, circulation, nerve body fluids, such as the metabolic system of participation, patients from resting to gradually restore again to limit state motion data, including heart rate, blood pressure, ecg analysis, such as traditional information, and provide important data with oxygen metabolism as the core, such as: peak VO_2_ and minute ventilation/ carbon dioxide production (VE/VCO_2_) slope are used to describe in detail the physiological conditions of patients in exercise state through rich and comprehensive monitoring indicators [76]. Discharged patients still need to pay attention to protection from viruses to avoid secondary infections. After recovery, they will be left with varying degrees of respiratory damage and lung dysfunction, which may cause other organ complications impacting daily life. It is recommended that during the home care period, suitable exercise rehabilitation videos can be utilized on multimedia equipment to insist on carrying out breathing rehabilitation training, such as breathing exercises, Tai Chi, eight-section brocade Chi Kung, yoga and other light exercise training [49,77]. With nutritional support, psychological counseling, and regular follow-up visits, symptoms such as dyspnea, shortness of breath, consolidate can be alleviated, which helps to improve and promote respiratory function, immune system, and exercise capacity. The subjects need to make full use of family, community, and medical resources for systematic and scientific rehabilitation training to lay a solid foundation for returning to society as soon as possible.

### 5.3. Remote Exercise Rehabilitation Methods for COVID-19 Patients

From the national incidence, about a quarter of the medical personnel are the direct victims of infectious disease. Epidemic prevention and control in the process of health care workers should pay attention to the cultivation of clinical thinking and judgment ability, concept not only stay on the disinfection, isolation and general knowledge, from the technical service extension to community service, and from the hospital service extension to the outside of the hospital service. The treatment and service will be placed in the same important position, penetration in the prevention, treatment, health care, rehabilitation and other aspects. Each country has also established the joint operation of “medical prescription” main attack and “exercise prescription” auxiliary attack. For example, the UK has established a national referral database and intervened in exercise prescription to strengthen the management and treatment of the national health condition [78]. The principles, content framework and minimum standard baseline for exercise for different populations were developed during the Australian pandemic [75]. Han University in the Netherlands has established an online service platform to provide the latest exercise prescription and programme support during the COVID-19 pandemic [79]. The United States has established a remote rehabilitation and testing system including COVID-19 physical and mental health and nursing services [80]. Japan makes use of TV, Internet, video and other technologies to produce the regular home version of the elderly’s physical function exercise prescription video [81]. Guidelines on physical activity based on social distance during COVID-19 in Korea [82]. The Chinese Association of Sports Science has scheduled to set up “Sports Health Promotion Centers” nationwide from 2020 to meet the public’s fitness needs and establish a library of traditional and popular exercise programs.

The majority of the COVID-19 disease outbreak affected the elderly, the majority of which have heart, brain, liver, kidney and other organs complication, and studies have confirmed that traditional project prevention, treatment and rehabilitation for the elderly COVID-19 have a positive effect [83]. It can promote the recovery of respiratory symptoms, deep, rhythmic breathing and body movement with the concentration of slow, make people relaxed mood, pressure drops into a state of meditation, priority is recommended to use qigong to prevent COVID-19 improve infection after respiratory symptoms [84]. Qigong can increase the number and activity of human immune cells, reduce inflammatory factors and inflammatory responses in the body, and train specific muscle groups to enhance physical strength through exercise [85,86]. Choosing Ba Duan jin, Tai Chi such delicate, smooth, soft, light movement, slow body movements and musculoskeletal stretch should be combined with body relaxation, deep breathing and mental concentration, emphasize the balance of the body and breath free, drives the whole body movement to increase the health of the body.

## 6. Future Directions

At present, China is in the post-epidemic prevention period, and some regions are in a state of sudden outbreak control. The construction of a COVID-19 prevention and control system has a long way to go. How to guide all people to resume physical exercise after being released from lockdowns and how to establish the stress mechanism of the whole population’s physical exercise during the major public health crisis has become an important research topic. First of all, based on scientific diagnosis of clinical treatment of COVID-19 in the fields of medicine and physiology, the direct correlation between physical exercise to improve immunity and enhance physical function and prevention and control of COVID-19 was systematically revealed, relevant intervention experiments were carried out, and a variety of physical exercise programs based on prevention or rehabilitation were developed. Second, it is necessary to improve the system and mechanism, promote the integration of physical exercise and medical cooperation to tackle the core technologies directly related to the treatment and rehabilitation of diseases, cross-boundary integration, promote the deep integration of technologies, resources and services of various systems, research and develop new measures, new models and new platforms for physical exercise intervention, and realize the intelligentization of national fitness services. Finally, science to build testing and support among family members, community and friends network support and guidance, health into the whole life cycle of the calendar year, inspire the inner motive power of physical exercise and vitality, the scientific and correct fitness methods and habits, cooperate with the public health system and professional platform of remote health services such as risk prevention and control system, ensure the effect of physical exercise. At present, about the movement that occupy the home monitoring and remote guide the study of risk prevention and control system, such as using modern technology to build system, accurate personalized exercise prescription for health promotion system and diversified remote intelligent security system, prevention and control of risk is a major public health crisis COVID-19 safeguard people’s key scientific movement that occupy the home. More and more relevant literatures have been published on “COVID-19 patient rehabilitation”. Through detailed and scientific implementation, it provides a theoretical basis for future clinical rehabilitation research in patients with similar respiratory diseases. At the same time, due to the need of isolation, prevention and control of most of the patients in the process of exercise rehabilitation after, use of remote medical treatment, remote service, wearable life feature detector, 5G technology, artificial intelligence, data analysis and other high-tech, of COVID-19 and other infectious diseases control and prevention, treatment and rehabilitation has an important value, broad prospects.

## Data Availability

Not applicable.
